# When Medical Management Is Not Enough: A Case Report of Progressive Thyromegaly Secondary to Hashimoto Thyroiditis

**DOI:** 10.7759/cureus.114335

**Published:** 2026-08-11

**Authors:** Kiana A Adams-Baker, Rebecca L Sanchez, Urmil Shukla, Jordana Paine

**Affiliations:** 1 Department of Applied Biomedical Sciences, University of the Incarnate Word School of Osteopathic Medicine, San Antonio, USA; 2 Department of General Surgery, Texas Surgery Center, Huntsville Memorial Hospital, Huntsville, USA; 3 Department of Pathology, Texas Surgery Center, Huntsville Memorial Hospital, Huntsville, USA

**Keywords:** autoimmunity, diffuse goiter, hashimoto's hypothyroidism, hashimoto thyroiditis, thyroid

## Abstract

A man in his mid-fifties with a more than 30-year history of hypothyroidism secondary to Hashimoto thyroiditis and a strong family history of thyroid disease was referred for a surgical evaluation after developing rapidly progressive thyromegaly associated with anterior neck discomfort, hoarseness, increasing neck fullness, and obstructive sleep apnea due to an enlarging neck circumference. His hypothyroidism had remained well controlled with daily levothyroxine therapy for decades, providing adequate biochemical control and symptomatic relief for his metabolic symptoms without prior evidence of significant thyroid enlargement. Despite this long-standing stability over a relatively short period following decades of clinical stability, he experienced an unexpected and rapid increase in thyroid size over a relatively short period, resulting in progressive compressive symptoms. Physical examination and imaging demonstrated an enlarged thyroid with compressive features to the adjacent cervical vasculature and structures, raising concerns for worsening airway compromise and other long-term compressive sequelae. Owing to the severity of the thyroid enlargement and compressive symptoms, it was determined that the best management was with surgical intervention. This case describes the unusual, delayed development of rapidly progressive compressive thyromegaly after more than three decades of stable Hashimoto thyroiditis managed with levothyroxine, an atypical clinical course that emphasizes the need for continued surveillance despite long-term biochemical control. Although hypothyroidism may remain metabolically controlled with thyroid hormone replacement, clinicians should remain vigilant for progressive structural thyroid disease and the development of compressive symptoms, as timely surgical intervention may be required.

## Introduction

Hypothyroidism secondary to Hashimoto thyroiditis commonly presents with metabolic symptoms such as fatigue, cold intolerance, brittle hair or hair loss, hypotension, and weight gain. Hashimoto thyroiditis is a prevalent autoimmune thyroid disorder, affecting approximately 10% of individuals in the United States, and demonstrates a strong female predominance. Diagnosis is supported by elevated levels of thyroid-stimulating hormone (TSH) and reduced levels of triiodothyronine (T3) and thyroxine (T4), accompanied by autoantibodies, most commonly thyroid peroxidase antibody (in 90% of cases), thyroglobulin antibody (in 50-80% of cases), and rarely TSH receptor antibody [1]. Histopathology demonstrates lymphocytic infiltration of the thyroid gland with follicular atrophy and the presence of Hurthle cells. The standard management of hypothyroidism includes thyroid hormone replacement therapy, such as levothyroxine [2].

The normal adult thyroid gland measures approximately 4.0 x 1.0 x 0.8 cm and weighs 20 to 25 grams. The thyroid gland increases with age and body size and is generally larger in males than in females. Enlargement beyond these expected dimensions is defined as thyromegaly [1]. Surgical management of thyromegaly is typically reserved for patients with symptomatic compressive symptoms secondary to thyromegaly or concern for malignancy. 

Although Hashimoto thyroiditis is more prevalent in women, men can also develop both metabolic symptoms of hypothyroidism and progressive thyroid enlargement resulting in compressive symptoms, a presentation that may be underrecognized. Levothyroxine remains the standard treatment for hypothyroidism associated with Hashimoto thyroiditis and is effective in restoring normal thyroid hormone levels; however, adequate biochemical and metabolic control does not necessarily prevent progressive thyromegaly [1]. This case illustrates the uncommon, delayed development of clinically significant compressive thyromegaly despite more than three decades of successful medical management. Clinicians should, therefore, continue to assess patients with Hashimoto thyroiditis for progressive neck enlargement and compressive symptoms during long-term follow-up, even when thyroid function is well controlled. Early recognition and shared decision-making regarding surgical referral are essential, as thyroidectomy may provide definitive symptom relief and prevent further compressive complications. 

## Case presentation

A male in his mid-50s with long-standing hypothyroidism secondary to Hashimoto thyroiditis was referred to a general surgery clinic due to progressive voice hoarseness, obstructive sleep apnea secondary to enlarging neck circumference, and localized anterior neck discomfort. He had been treated with levothyroxine sodium 50 mcg daily for the past thirty years. Over the preceding years, he noted progressive neck enlargement, worsening hoarseness, difficulty breathing with laying supine, increasing anterior neck discomfort, and reduced neck range of motion. 

His family history was notable for a strong predisposition to thyroid disease, as his father and two sisters have hypothyroidism secondary to Hashimoto thyroiditis. None of the stated family members with Hashimoto thyroiditis had experienced thyroid swelling or compressive symptoms. His medical history is remarkable for arthritis, hypercholesterolemia, hypothyroidism, obstructive sleep apnea, and diverticulosis. Current medications include levothyroxine sodium 50 mcg, atorvastatin calcium 40 mg, and meloxicam 5 mg, as well as nightly continuous positive airway pressure (CPAP) therapy. Patient had no allergies. 

At the initial diagnosis thirty years prior, laboratory findings demonstrated a markedly elevated thyroglobulin antibody level of 1189.4 IU/mL (reference range: 0-0.9 IU/mL) and an elevated TSH level of 7.03 uIU/mL (reference range: 0.45-4.5 uIU/mL). Over the course of treatment with levothyroxine, serial TSH levels revealed fluctuating biochemical control, ranging from 1.93 to 7.54 μIU/mL across several years. 

On physical examination, the thyroid gland was diffusely and bilaterally enlarged, without evidence of cervical lymphadenopathy. Given the progressive compressive symptoms, evidence of lymphadenopathy, and gland enlargement on examination, thyroid ultrasonography and computed tomography imaging of the neck and chest were obtained for further evaluation.

Investigations

**Figure 1:**
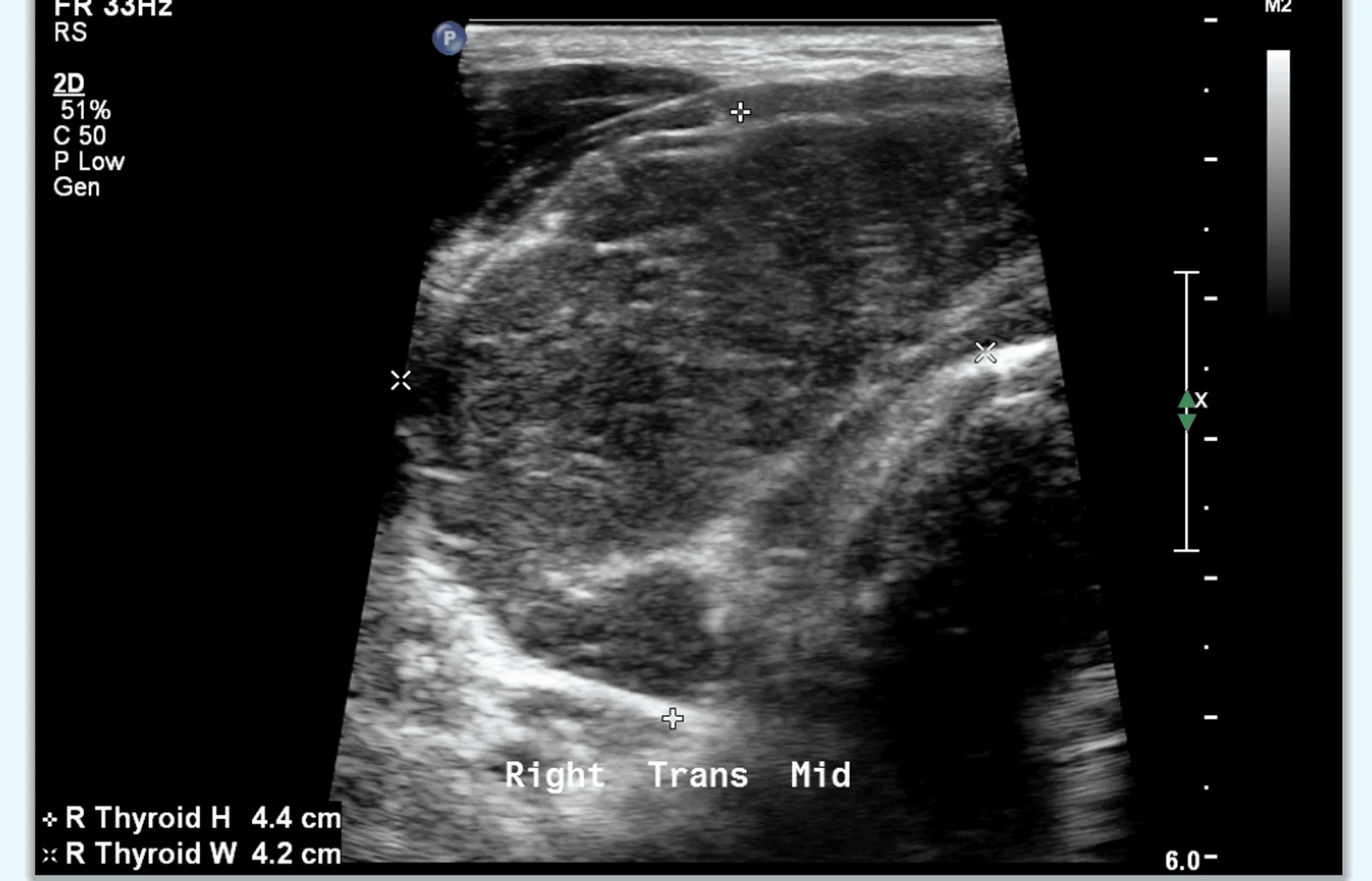
Transverse ultrasound view of the right lobe of the thyroid, measuring 4.4 x 4.2 cm.

**Figure 2:**
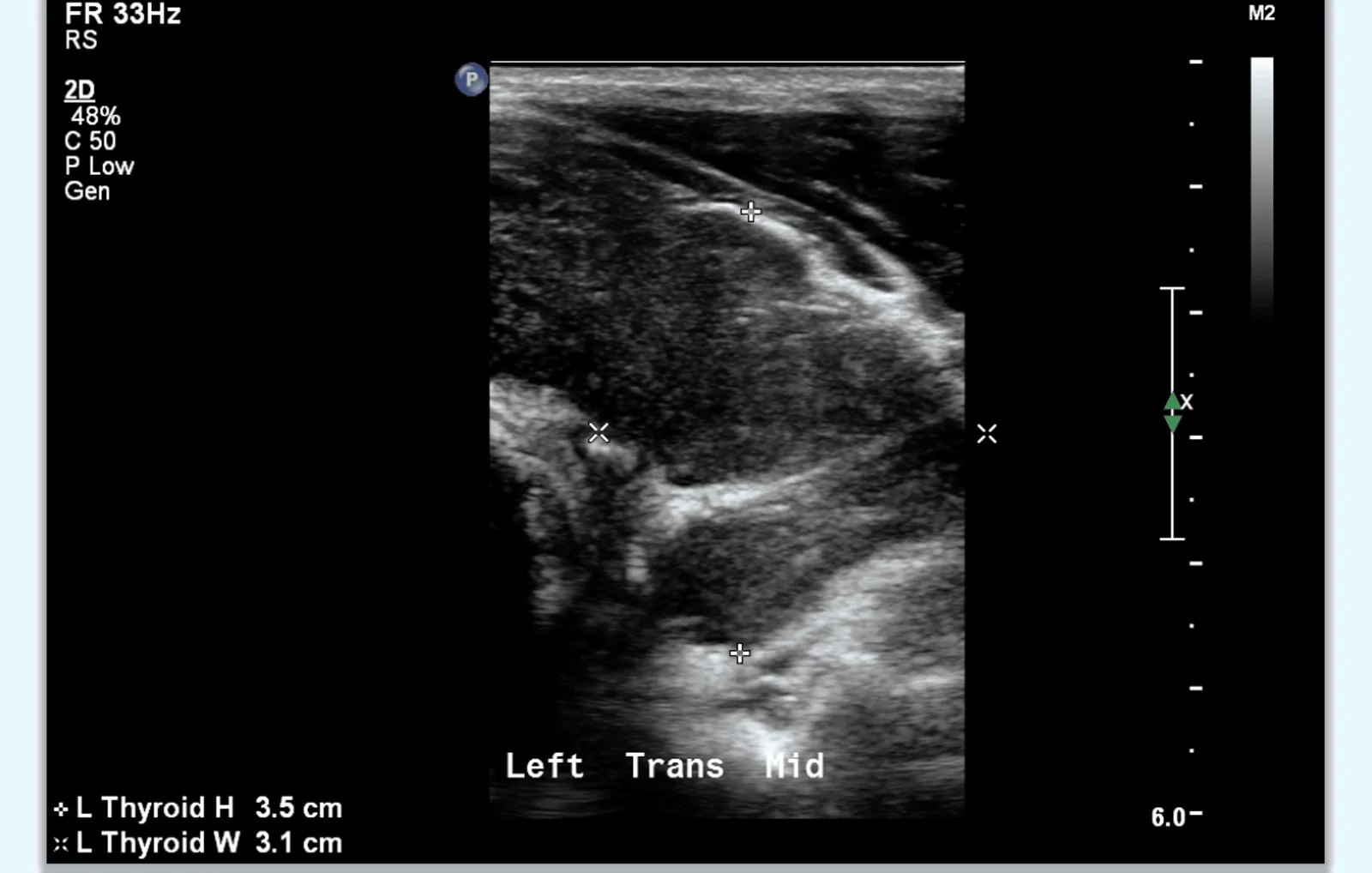
Transverse ultrasound view of the left lobe of the thyroid, measuring 3.5 x 3.1 cm.

**Figure 3:**
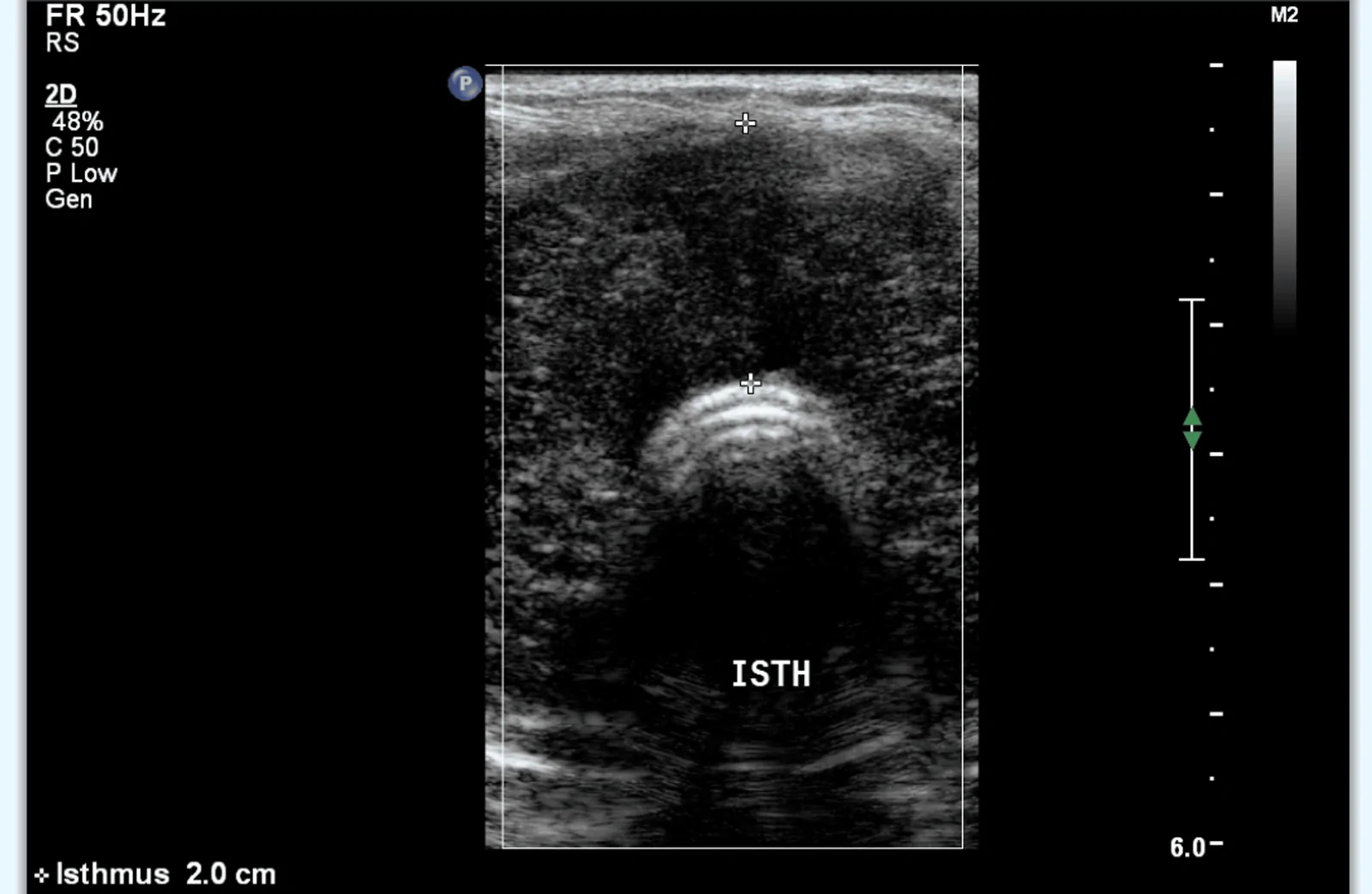
Transverse ultrasound view of the thyroid isthmus, measuring 2.0 cm.

Thyroid ultrasonography demonstrated diffused enlargement of the thyroid gland, with the measurements of the thyroid being right lobe 4.4 x 4.2 cm, left lobe 3.5 x 3.1 cm, and isthmus 2.0 cm. No thyroid nodules were identified (Figures 1-3). 

**Figure 4:**
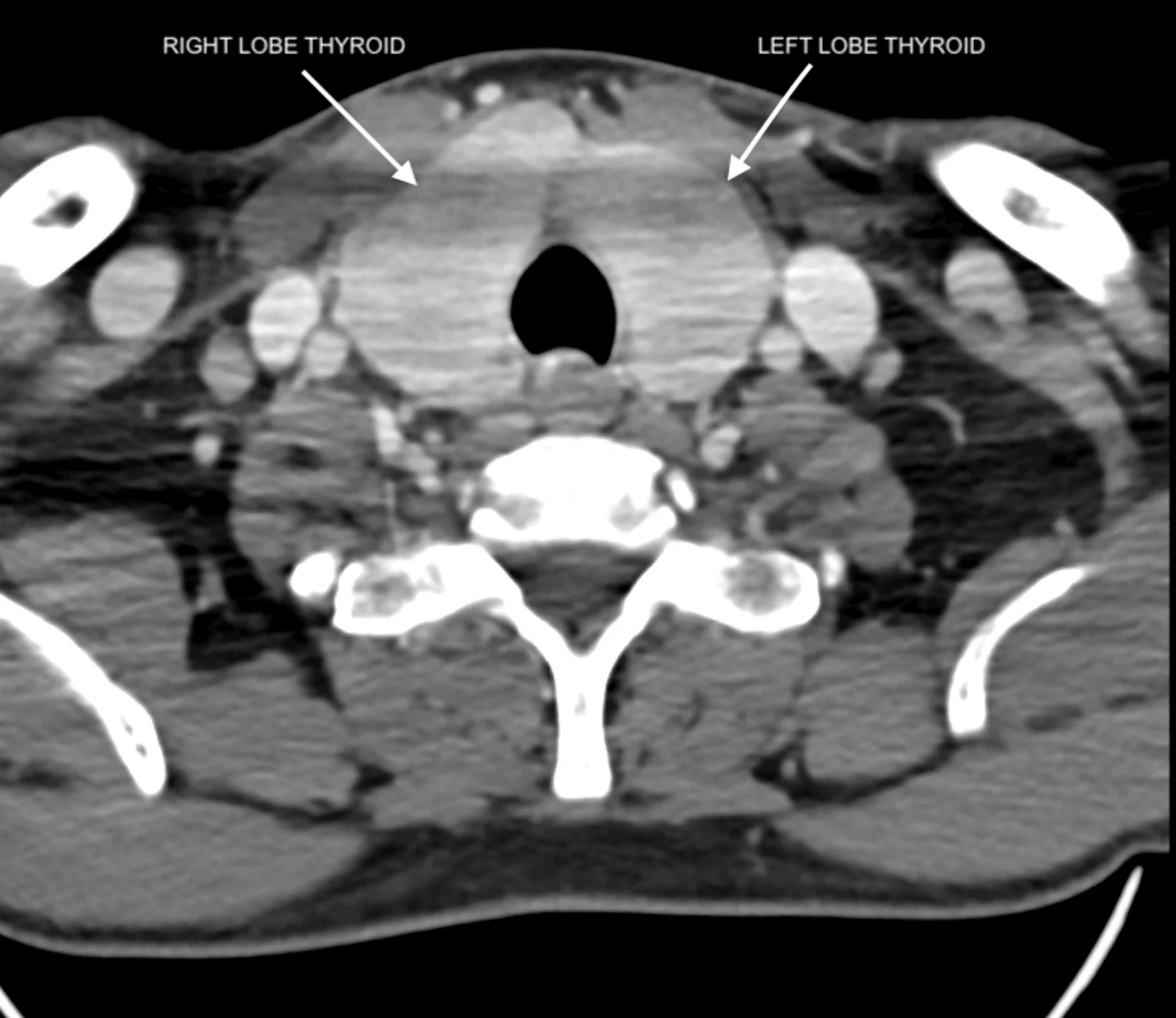
Contrast-enhanced axial CT of the chest with thyroid dimensions of 7.6 × 5.2 cm in transverse diameter and 7.9 cm craniocaudally.

Contrast-enhanced computed tomography of the chest was obtained to evaluate retrosternal extension and intrathoracic compression. Imaging revealed diffuse enlargement of the thyroid gland without a discrete parenchymal lesion, with maximal dimensions of 7.6 × 5.2 cm in transverse diameter and 7.9 cm craniocaudally. No intrathoracic abnormalities were identified (Figure 4).

In the office, fine needle aspiration (FNA) of the thyroid was obtained and revealed benign thyroid tissue with no evidence for malignancy. These investigations confirmed diffuse thyroid enlargement without nodular disease or evidence of malignancy, supporting the diagnosis of thyromegaly secondary to hypothyroidism.

Treatment

Following discussion about the presenting symptoms and review of the imaging and cytological results, the mutual agreement between the patient and physician was to proceed with total thyroidectomy for symptomatic compressive goiter. The surgical procedure, including its benefits and risks such as recurrent laryngeal nerve injury, hypocalcemia, and postoperative neck hematoma, was discussed in detail, and informed consent was obtained.

Surgery was scheduled for the following weeks, with a planned overnight post-operative stay for observation for airway compromise, incision site management, and hypocalcemia.

Outcome and follow-up

**Figure 5:**
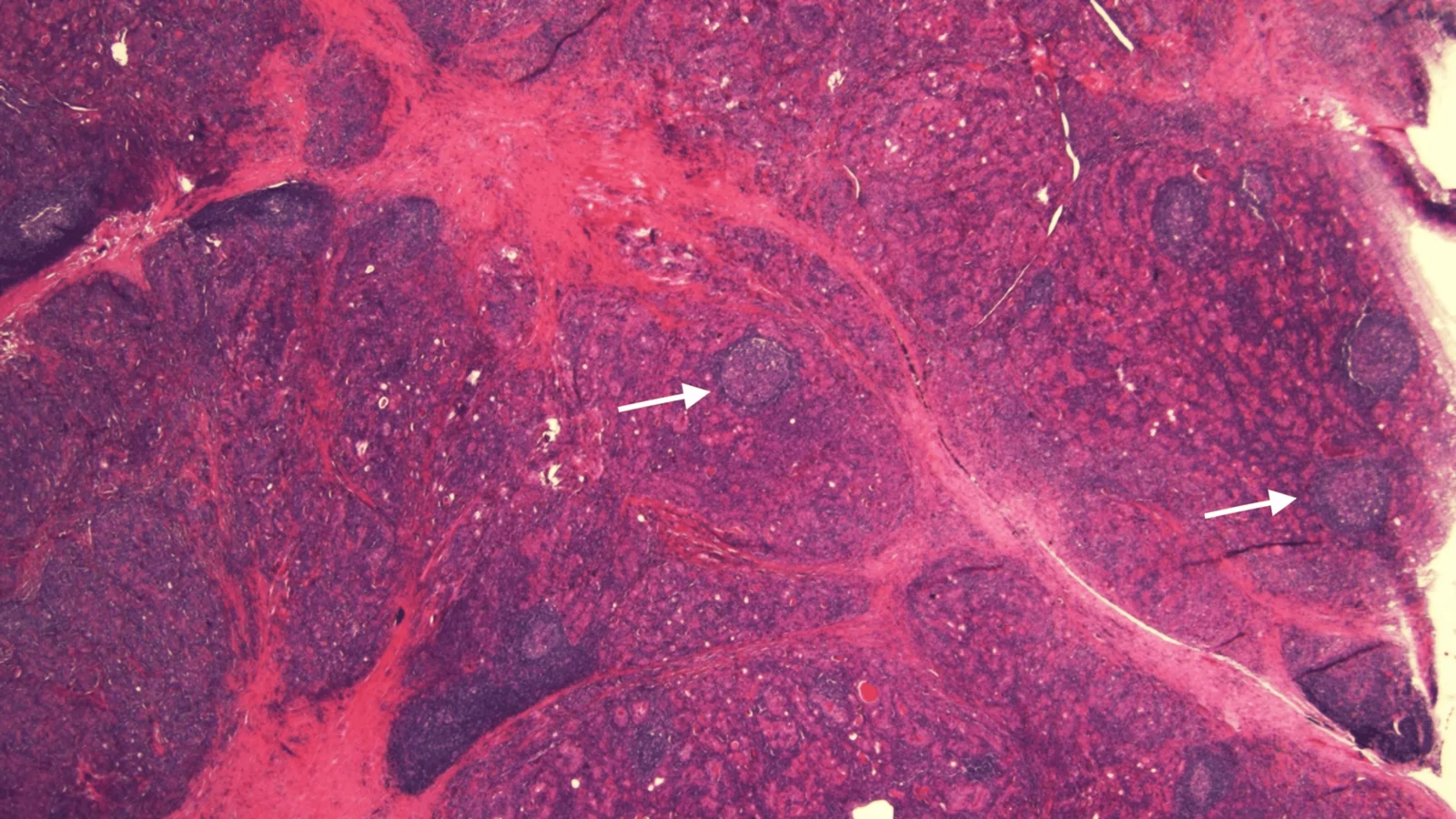
Histopathologic findings of the patient’s thyroid at 200x. Hashimoto thyroiditis is characterized by a dense lymphoplasmacytic infiltrate with scattered germinal centers (arrows) and increasing fibrosis over time.

**Figure 6:**
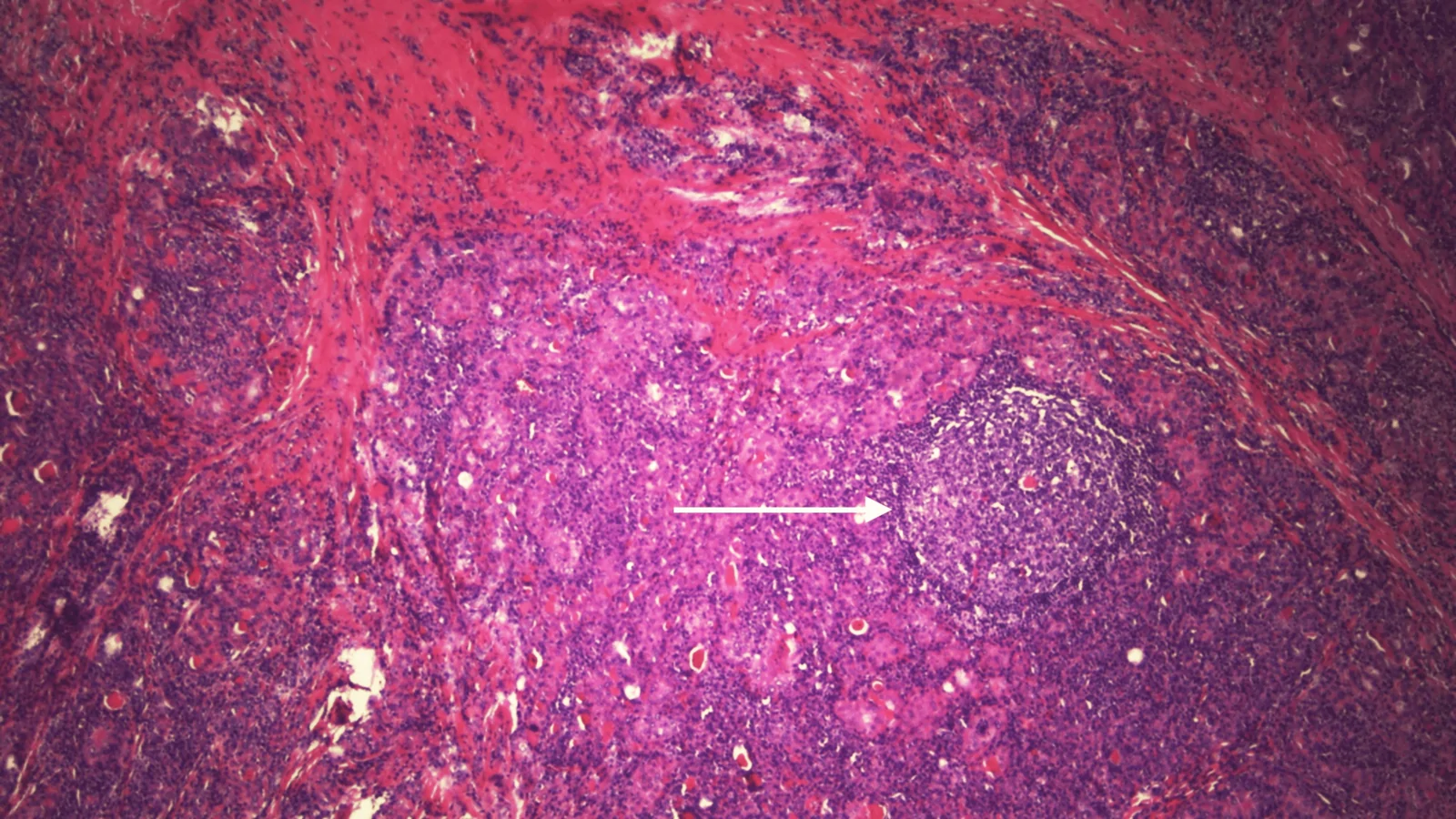
Histopathologic findings of the patient’s thyroid at 100x. Hashimoto thyroiditis is characterized by a dense lymphoplasmacytic infiltrate with scattered germinal centers (arrow) and increasing fibrosis over time.

**Figure 7:**
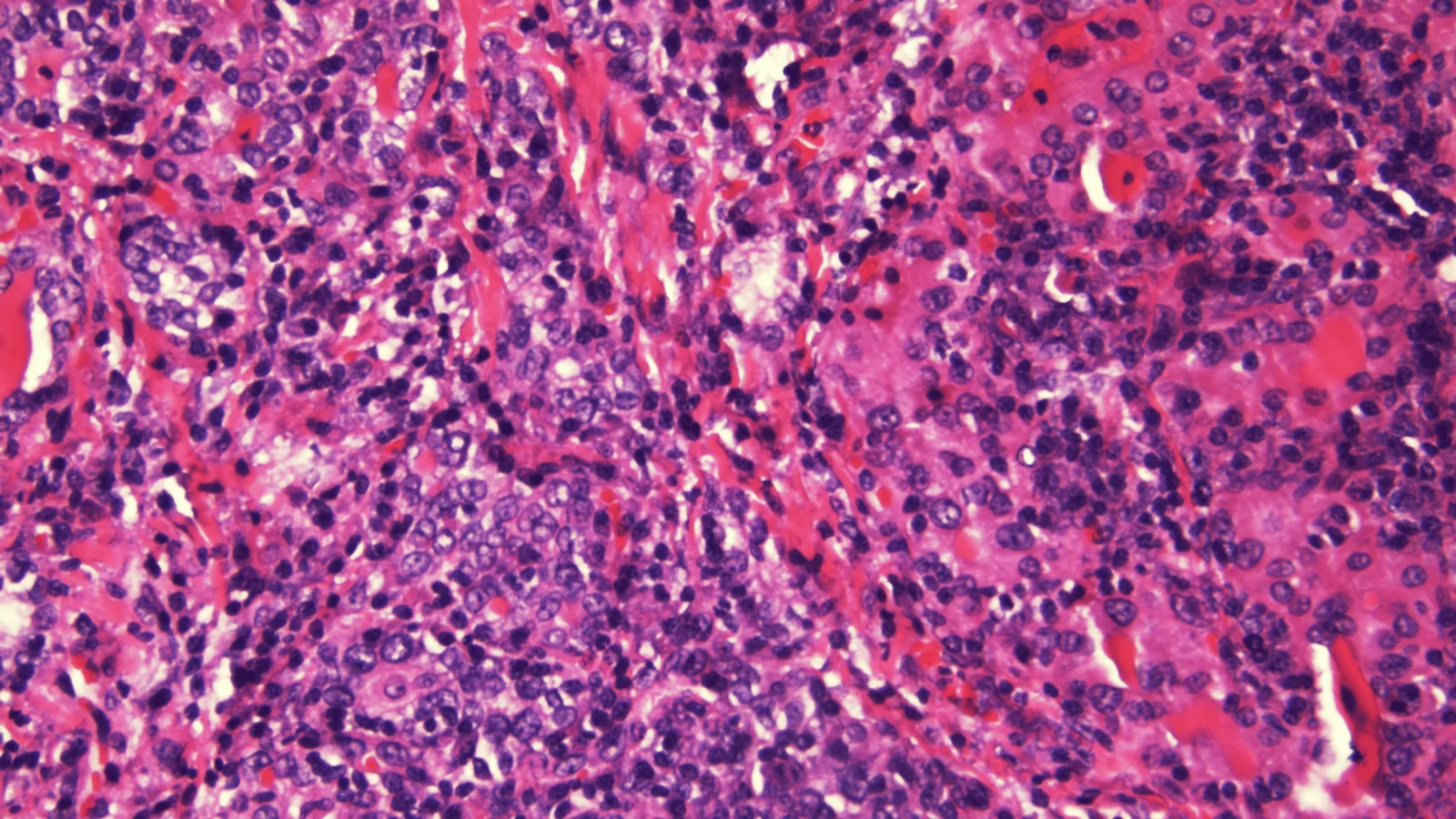
Histopathologic findings of the patient’s thyroid at 400x magnification. Hurthle cell change is also a prominent feature of Hashimoto thyroiditis, shown here by the follicular cells on the right half of the image with eosinophilic (pink) cytoplasm and variably sized nuclei in comparison to the normal residual follicular epithelial cells on the left half of the image.

**Figure 8:**
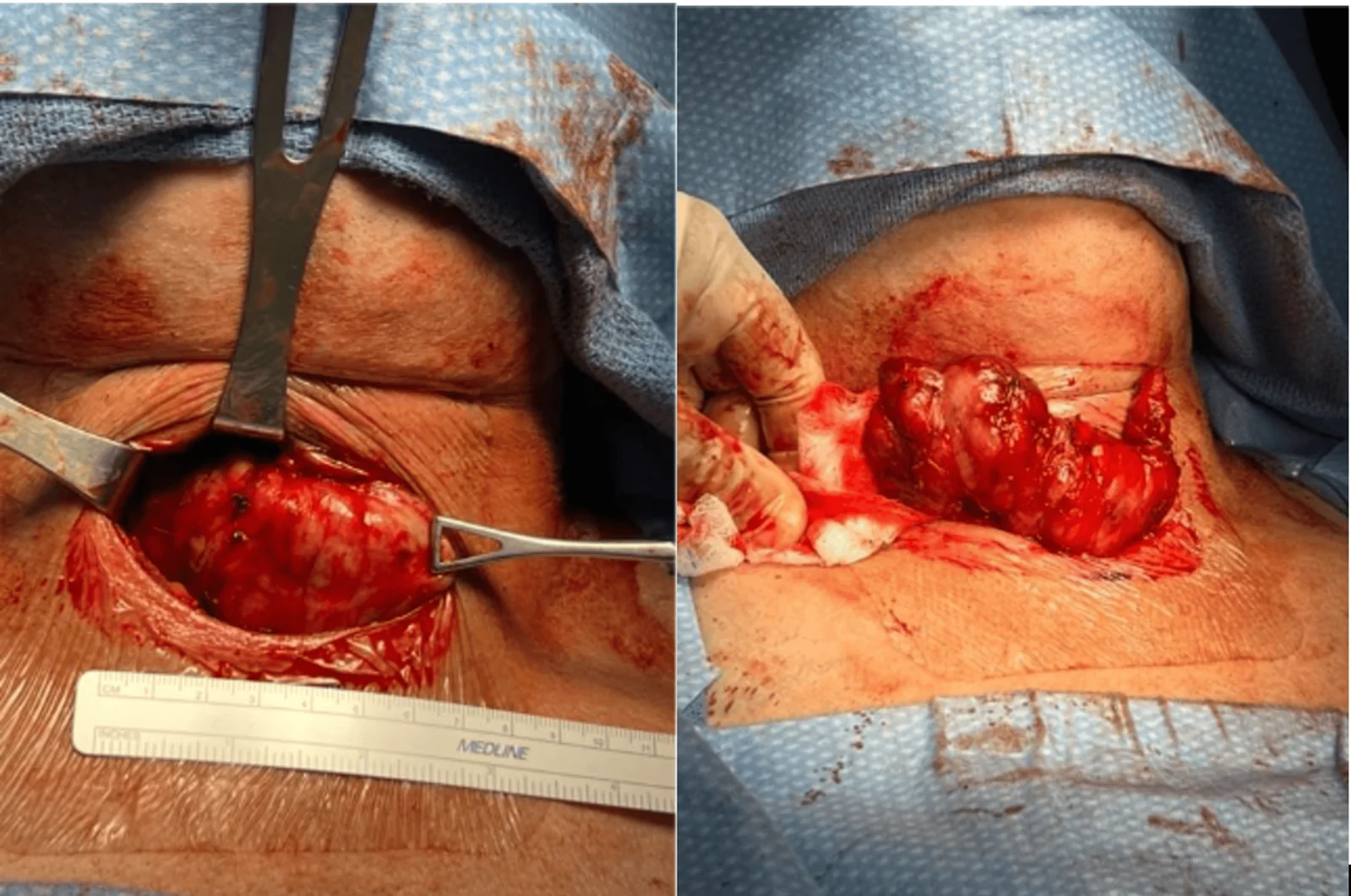
Intraoperative right lobe of thyroid measuring 6.5 cm in length.

Following total thyroidectomy, the resected thyroid tissue was sent to pathology for further evaluation. Pathology confirmed chronic lymphocytic thyroiditis in both lobes (Figures 5-7). Intraoperative measurement of the thyroid gland measured 6.5 cm in length (Figure 8). The pathology reports of the resected right lobe of the thyroid measured 10.0 × 6.5 × 2.5 cm, weighing 54.0 g, and the left lobe of the thyroid measured 8.6 × 5.0 × 2.6 cm, weighing 48.0 g.

Closed-suction drains were placed intraoperatively to reduce the risk of postoperative neck hematoma and removed on postoperative day three without complication. The patient was closely monitored for symptoms of hypocalcemia due to the removal of the parathyroids. Serum calcium remained within normal limits at 8.9 mg/dL (reference range: 8.5-10 mg/dL), and the patient reported no symptoms of hypocalcemia. 

Recovery was uncomplicated. The patient reported hoarseness that improved daily and remained on thyroid hormone replacement therapy. During the postoperative visit two weeks later in the in-office clinic, the incisions were healing well, and staples were removed. The patient was advised to avoid sun exposure for at least two months and apply a topical ointment for scar care. Patient continued to report hoarseness as his main symptom, requiring referral to a voice specialist, which improved after several months. He no longer utilizes the CPAP machine due to improvement of the obstructive sleep apnea, remains on thyroid hormone replacement (levothyroxine 75 mcg), and all thyroid hormone levels are within normal limits. The patient was encouraged to follow up with his primary care physician for further thyroid hormone level management. 

## Discussion

Many patients with Hashimoto thyroiditis develop hypothyroidism and present clinically with metabolic symptoms such as fatigue, hypotension, cold intolerance, brittle hair or hair loss, and weight gain. Medication management is the typical treatment with lifelong thyroid replacement on levothyroxine. However, some patients may also develop enlargement of the thyroid, resulting in compressive symptoms that may require surgical intervention, especially when malignancy cannot be excluded. In addition, Hashimoto thyroiditis is associated with an increased risk of developing papillary thyroid cancer [3]. According to the American Thyroid Association, surgical management is recommended for goiters with compressive symptoms, which include dyspnea, orthopnea, neck discomfort, and dysphagia [4]. In a study with over 260 patients who underwent a thyroidectomy for a range of thyroid diseases, only seven of the patients were found to have Hashimoto thyroiditis, utilizing the criteria of clinical testing and laboratory studies, in which cervical compression was rarely the indication for thyroidectomy [5].

This case describes a male in his mid-50s with a long-standing history of Hashimoto thyroiditis who developed progressively worsening compression symptoms. This presentation is clinically notable given the strong female predominance of Hashimoto thyroiditis. Similar cases have been reported in the literature; however, they remain uncommon. Ogrin et al. reported a male in his early 50s who had been diagnosed with hypothyroidism due to Hashimoto thyroiditis who soon developed cervical compression with the Pemberton sign (venous obstruction) within two months of diagnosis. This patient eventually underwent total thyroidectomy due to worsening neck discomfort, venous obstruction, and breathing difficulties [6]. Similarly, Babaya et al. reported a male in his late 50s who presented to the clinic with an enlarged goiter and hoarseness, who was diagnosed with Hashimoto thyroiditis. The patient was managed with thyroid replacement initially, but later proceeded with a total thyroidectomy due to the compressive nature, with an eventual diagnosis of malignancy following histological evaluation of the resected thyroid [7]. These cases, together with the present case, underscore the importance of recognizing progressive thyroid enlargement and compressive symptoms in male patients, which ultimately led to surgical management (Table 1). The patient’s family history further supports the recognized strong genetic predisposition associated with autoimmune chronic lymphocytic thyroiditis. 

**Table 1:** Clinical cases of patients diagnosed with hypothyroidism due to Hashimoto thyroiditis.

Study	Age/Sex	Presentation	Thyroid findings	Management	Outcome
Ogrin et al. [6]	Early 50s M	Neck discomfort, compressive symptoms	Diffuse Hashimoto's goiter	Total thyroidectomy	Symptom resolution
Babaya et al. [7]	Late 50s M	Hoarseness, thyroid enlargement	Hashimoto thyroiditis with malignancy	Total thyroidectomy	Good recovery
Present case	Mid-50s M	Hoarseness, anterior neck discomfort, ↓ range of motion (ROM)	Diffuse Hashimoto's goiter	Total thyroidectomy	Symptom improvement

Evaluation of Hashimoto thyroiditis integrates clinical assessment with laboratory, imaging, and histopathological results. The diagnosis is further supported by autoantibodies and abnormal thyroid hormone levels. In this case, the patient initially presented with mild symptoms of hypothyroidism, including fatigue, and was found to have elevated antibodies to thyroid peroxidase and fluctuating TSH levels, confirming the diagnosis of Hashimoto thyroiditis. Ultrasonography and CT imaging revealed diffuse glandular enlargement without distinct nodules or intrathoracic extension. FNA is a valuable diagnostic tool for differentiating between benign and malignant features, given the association with Hashimoto thyroiditis and papillary thyroid carcinoma. Collectively, findings supported a benign thyromegaly with lymphocytic infiltrates, which was further supported by histology following his thyroidectomy. 

Surgical intervention was pursued due to the patient’s progressive and clinically significant symptoms, including voice hoarseness, sleep apnea due to an enlarging neck circumference, and decreased range of motion. Despite long-term medical management with thyroid hormone replacement therapy and biochemical control, the progressive structural enlargement and associated symptoms warranted consideration of surgical management. Total thyroidectomy was performed by general surgery, with favorable postoperative outcomes. Histopathology confirmed chronic lymphocytic infiltrates within the thyroid parenchyma without evidence of malignancy. Postoperative risks, including recurrent laryngeal nerve injury, hypocalcemia, and postoperative neck hematoma, were assessed and monitored. The patient experienced hoarseness, which was treated and followed by a vocal specialist, and exhibited no evidence of hypocalcemia or neck hematoma. 

This case highlights that, despite the noteworthy female predominance, Hashimoto thyroiditis can produce clinically significant progressive thyromegaly with associated compression. Adequate biochemical and metabolic control with hormone replacement therapy does not always necessarily prevent structural thyroid changes. Therefore, physicians should remain vigilant for the development of compressive symptoms secondary to Hashimoto thyroiditis, including in men, as progressive symptomatic thyromegaly may warrant further treatment and management with surgical intervention to provide symptomatic relief. 

## Conclusions

This case describes an uncommon presentation of Hashimoto thyroiditis characterized by delayed, progressive compressive thyromegaly despite more than three decades of adequate levothyroxine therapy and stable thyroid function tests. While thyroid hormone replacement effectively manages the metabolic consequences of hypothyroidism, it does not always prevent structural progression of autoimmune thyroid disease. Recognition of progressive thyroid enlargement and associated compressive symptoms remains essential, as timely surgical evaluation may provide definitive symptom relief and prevent further complications. This case also highlights the importance of considering significant thyroid enlargement in male patients, a population less commonly affected by Hashimoto thyroiditis and potentially at risk for delayed recognition of symptomatic disease. 
